# Autophagy–NAD^+^ axis: emerging insights into neuronal homeostasis and neurodegenerative diseases

**DOI:** 10.3389/fmolb.2025.1695486

**Published:** 2025-11-26

**Authors:** Gamze Kocak, Mylla M. Dimas, Luiz F. S. E. Silva, Danyelle Silva-Amaral, Congxin Sun, Sophie Cruddas, Timothy Barrett, Daniel Martins-de-Souza, Edecio Cunha-Neto, Patricia S. Brocardo, Tetsushi Kataura, Viktor I. Korolchuk, Sovan Sarkar

**Affiliations:** 1 Department of Cancer and Genomic Sciences, School of Medical Sciences, College of Medicine and Health, University of Birmingham, Birmingham, United Kingdom; 2 Department of Morphological Sciences, Centre of Biological Sciences, Federal University of Santa Catarina, Florianopolis, Santa Catarina, Brazil; 3 Laboratory of Immunology, Heart Institute (InCor), and Division of Clinical Immunology and Allergy, Hospital das Clínicas da Faculdade de Medicina da Universidade de São Paulo, São Paulo, Brazil; 4 Institute for Investigation in Immunology (III), Institutos Nacionais de Ciências e Tecnologias (INCT), São Paulo, Brazil; 5 Laboratory of Neuroproteomics, Institute of Biology, University of Campinas, Campinas, Brazil; 6 Department of Endocrinology, Birmingham Women’s and Children’s Hospital, Birmingham, United Kingdom; 7 Experimental Medicine Research Cluster (EMRC), University of Campinas, Campinas, Brazil; 8 D’Or Institute for Research and Education (IDOR), São Paulo, Brazil; 9 INCT in Modelling Human Complex Diseases With 3D Platforms (Model3D), Conselho Nacional de Desenvolvimento Científico e Tecnológico, São Paulo, Brazil; 10 Department of Neurology, Institute of Medicine, University of Tsukuba, Tsukuba, Japan; 11 Biosciences Institute, Faculty of Medical Sciences, Newcastle University, Newcastle upon Tyne, United Kingdom

**Keywords:** autophagy, NAD^+^, NAD^+^ precursor, NAD^+^-dependent enzyme, neuroprotection, neuronal cell death, autophagy inducer, NAD^+^ supplementation

## Abstract

Autophagy is an evolutionarily conserved catabolic process that plays a central role in maintaining cellular homeostasis by degrading and recycling damaged or surplus proteins, organelles, and other cellular macromolecules and components. A growing body of evidence highlights a bidirectional relationship between autophagy and nicotinamide adenine dinucleotide (NAD^+^), a vital metabolic cofactor involved in numerous cellular processes, including energy metabolism, genomic maintenance, stress resistance, and cell survival. Autophagy supports NAD^+^ homeostasis by recycling metabolic precursors, while NAD^+^-dependent enzymes such as sirtuins and PARPs regulate autophagy initiation and lysosomal function. Disruption of this autophagy–NAD^+^ axis has emerged as a common feature in several neurodegenerative diseases, where impaired cellular clearance and metabolic dysfunction contribute to neuronal vulnerability. In this review, we summarize the advances of the molecular links between autophagy and NAD^+^ metabolism, with a particular focus on their roles in mitochondrial quality control, bioenergetic regulation, and cellular resilience. We also discuss the therapeutic potential of targeting the autophagy–NAD^+^ axis to promote neuroprotection in neurodegenerative disease.

## 1 Introduction

Maintaining cellular homeostasis is essential to the physiology of an organism. Macroautophagy (henceforth, autophagy) is among the main catabolic processes by which recycling of cellular macromolecules and organelles is carried out. This process involves a double-membraned vesicle called autophagosome engulfing the autophagic cargo, and its subsequent fusion with the lysosome to mediate cargo digestion (Yamamoto et al., 2023). Increasingly, dysregulation of autophagy is being linked to ageing and the pathology in a myriad of human diseases, including several neurodegenerative and lysosomal storage diseases (Seranova et al., 2017; 2020; Aman et al., 2021; Nixon and Rubinsztein, 2024). Post-mitotic neuronal cells are particularly vulnerable to autophagy malfunction due to their inability to clear toxic protein aggregates and damaged mitochondria, and thus, failure to maintain autophagy-mediated cellular homeostasis in neurons contributes to neurodegeneration (Palmer et al., 2025).

**FIGURE 1:**
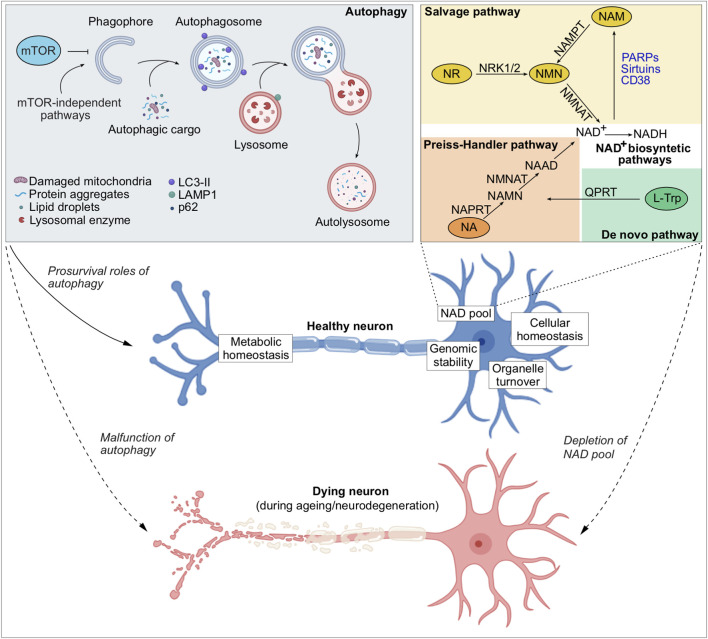
Autophagy and NAD^+^ metabolism in neuronal health and disease. Schematic overview illustrating the interplay between autophagy and NAD^+^ metabolism in maintaining neuronal homeostasis. Autophagy can be initiated through mTOR-dependent or mTOR-independent pathways, driving the formation of phagophores that elongate and capture autophagic cargo (such as damaged organelles, protein aggregates, lipid droplets) to form autophagosomes. The autophagosomes subsequently fuse with the lysosomes to degrade the autophagic cargo, thereby ensuring organelle turnover and recycling of the macromolecular breakdown products to aid metabolic homeostasis. Apart from cellular macromolecules and organelles, autophagy maintains intracellular NAD pool. NAD^+^ levels are sustained through multiple biosynthesis pathways, including the salvage, the Preiss–Handler, and *de novo* pathways. Among these, the salvage pathway predominates, recycling NAM (a by-product of NAD^+^-consuming enzymes such as sirtuins, PARPs, and CD38) back into NAD^+^. A healthy neuron is maintained by adequate autophagy and intracellular NAD^+^ levels, maintaining metabolic and cellular homeostasis, genomic stability, and efficient organelle turnover. In contrast, malfunction of autophagy or depletion of the NAD pool during ageing and neurodegeneration leads to neuronal cell death.

Autophagy is a multistep, tightly regulated pathway initiated by the formation of a phagophore, a cup-shaped membrane that elongates and engulfs cargo destined for degradation (Figure 1). This nascent autophagosome requires the coordinated action of autophagy-related (ATG) proteins, including the ULK1 initiation complex, the class III phosphatidylinositol 3-kinase (PI3K) complex, and two ubiquitin-like conjugation systems that mediate lipidation of ATG8/LC3 proteins onto the growing membrane (Yamamoto et al., 2023). Autophagosomes can capture bulk cytosolic material, but they also selectively target damaged or superfluous organelles, aggregated proteins, or invading pathogens, a process often mediated by selective autophagy receptors such as p62/SQSTM1, NBR1, NDP52, and OPTN, which bridge ubiquitinated cargo to LC3 (Vargas et al., 2023). Once sealed, the mature autophagosome traffics along the cytoskeleton and fuses with lysosomes, generating autolysosomes in which the cargo is degraded by acidic hydrolases. The resulting macromolecular building blocks, including amino acids, fatty acids, and nucleotides, are recycled back into the cytoplasm to support biosynthesis and energy production. Autophagy is under dynamic regulation by nutrient and stress signalling pathways, with mTORC1 acting as a key negative regulator and AMPK as an additional modulator, thereby coupling autophagy induction to cellular energy status (Rabanal-Ruiz et al., 2017). In neurons, selective forms of autophagy such as mitophagy play a particularly critical role in preserving organelle quality and bioenergetic competence (Nixon and Rubinsztein, 2024). Thus, autophagy integrates degradative capacity with metabolic flexibility, and its impairment not only compromises proteostasis and organelle integrity but also undermines cellular adaptation to stress, contributing to the pathogenesis of age-related and neurodegenerative disorders (Palmer et al., 2025).

Beyond its canonical degradative function, autophagy has emerged as a central regulator of cellular metabolism linked to cell survival. Recent studies have identified a pro-survival role of autophagy by maintaining intracellular levels of a metabolic cofactor, nicotinamide adenine dinucleotide (NAD^+^). Loss of autophagy leads to depletion of NAD^+^ that mediates cytotoxicity in an evolutionarily conserved mechanism from yeast cells to human neurons (Kataura et al., 2022; Sun et al., 2023). Consistent with these findings, NAD^+^ functions as a cofactor for a wide array of redox and non-redox enzymes that facilitate energy metabolism, DNA repair, chromatin remodelling, and other biological processes, thereby playing a key role in maintaining genomic stability and cellular health (Covarrubias et al., 2021). In cells, NAD^+^ biosynthesis occurs *via* three main pathways: the *de novo* pathway from tryptophan, the Preiss-Handler pathway from nicotinic acid, and the salvage pathway from nicotinamide (Figure 1); the latter being most utilized by the cells (Katsyuba et al., 2020; Covarrubias et al., 2021). In the salvage pathway, the NAD^+^ biosynthetic enzymes include nicotinamide phosphoribosyl transferase (NAMPT) and nicotinamide mononucleotide adenylyl transferases (NMNATs) (Cambronne and Kraus, 2020). NAMPT converts NAM to NMN, which is then converted to NAD^+^ by NMNATs. The NMNAT isoforms—NMNAT1, NMNAT2 and NMNAT3—are respectively localised in the nucleus, cytoplasm and mitochondria, and enable subcellular control of NAD^+^ synthesis (Berger et al., 2005). The localisation of NAMPT and NMNAT3 in mitochondria indicates the existence of an intrinsic mitochondrial NAD^+^-salvage pathway critical for sustaining mitochondrial NAD^+^ levels and bioenergetic function (Berger et al., 2005; Nikiforov et al., 2011; Wang X. et al., 2019). Apart from the roles of NAD^+^ biosynthetic enzymes, NAD^+^-dependent enzymes (PARPs, SIRTs, CD38/CD157, and SARM1) can directly regulate autophagy and mitochondrial quality control in a context-dependent manner, linking metabolic state to degradative pathway regulation (Fang et al., 2016; Wilson et al., 2023).

Together, multiple lines of evidence delineate an intricate bidirectional interplay between autophagy and NAD^+^ metabolism that ensures cellular homeostasis under metabolic stress. Moreover, both autophagy and NAD^+^ levels are necessary for mitochondrial turnover and function, which are crucial for neuronal homeostasis and survival (Fang et al., 2016; Kataura et al., 2024). Of biomedical relevance, pharmacological strategies enhancing autophagy or boosting NAD^+^ levels show therapeutic benefits in neurodegenerative disease models (Menzies et al., 2017; Lautrup et al., 2019). Therefore, the autophagy–NAD^+^ axis represents a promising therapeutic avenue for ageing and neurodegenerative diseases that are characterized by progressive decline in autophagic activity and NAD^+^ levels (Wilson et al., 2023). In this review, we focus on the significance of the bidirectional relationship between autophagy and NAD^+^, with a particular emphasis on the role of autophagy–NAD^+^ axis in cellular, mitochondrial and bioenergetic homeostasis in neurons, and its implications and therapeutic opportunities in neurodegenerative diseases.

## 2 The link between autophagy and NAD^+^ in neurons

Autophagy or NAD^+^ homeostasis have been shown to independently support cellular health across species, from yeast to mice (Katsyuba et al., 2020; Aman et al., 2021; Covarrubias et al., 2021; Wilson et al., 2023; Yamamoto et al., 2023). Growing evidence suggests a bidirectional relationship between autophagy and NAD^+^, where NAD^+^ replenishment can normalize cellular function by modulating autophagy (Fang et al., 2014; 2016; 2019a; Kataura et al., 2024), whilst activation or restoring autophagy can improve NAD^+^ levels across various cell types (Desquiret-Dumas et al., 2013; Alshawi and Agius, 2019; Zhang et al., 2020; Kataura et al., 2024). Our recent findings reveal that autophagy plays a vital role in maintaining intracellular NAD pool (Kataura et al., 2022; Sun et al., 2023). The mechanisms underlying this reciprocal relationship, especially in neuronal homeostasis, are not fully understood. We will further discuss about this link in the context of mitochondrial and bioenergetic homeostasis, as well as overall cellular homeostasis, in neurons.

### 2.1 Autophagy–NAD^+^ axis in maintaining cellular homeostasis in neurons

Autophagy and NAD^+^ are vital for neuronal homeostasis and survival, whereas their deficits are associated with ageing and neurodegeneration (Sedlackova and Korolchuk, 2020; Schmauck-Medina et al., 2022; Wilson et al., 2023) (Figure 1). Studies on the molecular roles of NAD^+^ in cellular physiology highlight autophagy as a key regulator—by preventing DNA damage leading to excessive NAD^+^ consumption through mitochondrial quality control, and by mitigating nutrient stress *via* the recycling of amino acids, lipids, and nucleosides (Sedlackova and Korolchuk, 2020). We recently demonstrated that autophagy plays a crucial role in supporting cellular survival by maintaining intracellular NAD^+^ levels (Kataura et al., 2022; Sun et al., 2023). This is pertinent for post-mitotic neurons which rely on functional autophagy for clearing undesirable cellular materials like protein aggregates and damaged mitochondria (Stavoe and Holzbaur, 2019). Genetic studies in mice have shown that inducible knockout of essential autophagy-related genes (*Atg5* or *Atg7*) in the brain leads to accumulation of protein aggregates and neurodegeneration (Hara et al., 2006; Komatsu et al., 2006), implying that basal autophagy is crucial for neuronal survival.

Emerging evidence highlights a crucial role for autophagy in maintaining neuronal homeostasis through the regulation of NAD^+^ metabolism. Studies employing autophagy-deficient models, including *ATG5* ^*−/−*^ human embryonic stem cells (hESC)-derived neurons and mouse embryonic fibroblasts, have revealed that loss of autophagy disrupts cellular metabolism and NAD^+^ homeostasis, leading to mitochondrial dysfunction, disrupted proteostasis, and increased cell death (Kataura et al., 2022; Sun et al., 2023). Mechanistically, these effects are driven by hyperactivation of NAD^+^-consuming enzymes (NADases) such as *poly-ADP ribose polymerases* (PARPs) and sirtuins (SIRTs), which deplete the total NAD pool. Notably, depletion of NAD^+^ and NADH triggered a cytotoxic cascade involving mitochondrial depolarization and oxidative stress, culminating in cell death (Kataura et al., 2022; Sun et al., 2023). This phenomenon was evolutionarily conserved from yeast to mammals (Kataura et al., 2022). In these autophagy-deficient models, boosting NAD^+^ levels by supplementation with NAD^+^ precursors such as nicotinamide (NAM), nicotinamide riboside (NR) or nicotinamide mononucleotide (NMN), or by preventing NAD^+^ consumption with PARP and SIRT inhibitors, restored mitochondrial function and bioenergetics, and improved cell viability (Kataura et al., 2022; Sun et al., 2023). Interestingly, NAD^+^ boosters also suppressed the buildup of aggresomes (accumulation of misfolded protein aggregates) that are typically observed in autophagy-deficient cells (Sun et al., 2023), highlighting a potential interplay between mitochondrial and protein homeostasis (Katsyuba et al., 2018; Ruan et al., 2020; Nowicka et al., 2021; Romani et al., 2021). These findings suggest the importance of the autophagy–NAD^+^ axis in neuronal survival.

### 2.2 Mitochondrial homeostasis in neuronal health

Beyond energy production, mitochondria are pivotal regulators of neuronal signalling and plasticity (Rangaraju et al., 2019). Maintaining mitochondrial homeostasis—or mitostasis, which encompasses mitochondrial function, distribution, and quality—is essential for neuronal health due to their unique morphology, extensive architecture, and high metabolic demands (Misgeld and Schwarz, 2017). A key aspect of mitostasis is mitophagy, which selectively eliminates damaged mitochondria (Pickles et al., 2018), thereby preventing oxidative stress and sustaining mitochondrial and cellular NAD^+^ levels (Sedlackova and Korolchuk, 2020; Kataura et al., 2024). The PINK1–Parkin pathway is a well-characterized mechanism in this process; PINK1 senses mitochondrial damage, while Parkin tags impaired mitochondria for autophagic clearance (Narendra and Youle, 2024).

Neurons mainly generate ATP through oxidative phosphorylation (OXPHOS), in contrast to glial cells that rely mainly on glycolysis (Zheng et al., 2016). Although the brain constitute only about 2% of body mass, it consumes about 20% of body’s oxygen to support energy-intensive functions, including action potentials, neurotransmission, synaptic vesicle recycling, and axonal transport (Raichle and Gusnard, 2002; Howarth et al., 2012). Glucose is first metabolized through glycolysis to pyruvate, which enters the tricarboxylic acid cycle (TCA) *via* pyruvate dehydrogenase. The TCA cycle generates NADH and FADH_2_, which fuel the mitochondrial electron transport chain (ETC) and ATP synthase (Martínez-Reyes and Chandel, 2020). Notably, neuronal metabolism is spatially compartmentalized: somata preferentially use aerobic glycolysis to limit ROS, whereas axon terminals depend on OXPHOS, preserving nuclear and cytoplasmic integrity (Wei et al., 2023).

Central to these mitochondrial functions is NAD^+^, cycles between oxidised (NAD^+^) and reduced (NADH) forms to drive electron transport and ATP production. NAD^+^ exists in compartmentalised pools—approximately 250 µM in the mitochondria, 100 µM in the cytoplasm, and in the nucleus—regulated by NAD^+^-consuming enzymes that modulate turnover in a compartment-specific manner (Cambronne et al., 2016). The NAD^+^ biosynthetic enzyme NAMPT, which progressively declines with ageing and neurodegeneration, is essential for maintaining mitochondrial and neuronal health; its loss disrupts mitochondrial homeostasis and causes neurodegeneration in mice (Wang et al., 2017; Lautrup et al., 2019; Shen et al., 2023; Chen et al., 2024). NAD^+^ precursor supplementation can rescue mitochondrial dysfunction, mitophagy, and alleviate disease-related phenotypes (Yoshino et al., 2018; Dölle and Tzoulis, 2025). These findings highlight the neuroprotective roles of NAD^+^, suggesting that its effects may be mediated through the enhancement of mitochondrial biogenesis, function and mitophagy.

Altogether, NAD^+^ availability is closely intertwined with mitochondrial health. A decline in NAD^+^ levels impair mitochondrial function, diminishes ATP production, and jeopardises neuronal viability (Lautrup et al., 2019; Wilson et al., 2023). Moreover, the PINK1–Parkin pathway and mitophagy intersect with NAD^+^-dependent signalling, underscoring the bidirectional crosstalk between mitochondria quality control and NAD^+^ homeostasis (Fang et al., 2016; Kataura et al., 2024). This precise spatial and metabolic regulation positions NAD^+^ as a central hub integrating mitochondrial homeostasis with autophagy-dependent neuronal survival.

### 2.3 NAD^+^-dependent enzymes in neuronal autophagy

Key NAD^+^-consuming enzymes—PARPs, SIRTs, CD38/CD157, and SARM1—coordinately regulate neuronal physiology and autophagy (Zhang D.-X. et al., 2016; Covarrubias et al., 2021; Wilson et al., 2023). Among these, SIRTs are NAD^+^-dependent deacetylases and mono-ADP-ribosyltransferases that control transcriptional activities and various cellular processes including DNA repair, mitochondrial biogenesis, and autophagy (Houtkooper et al., 2012; Ng and Tang, 2013; Wu et al., 2022; Baeken, 2024). SIRT1, SIRT2, and SIRT3 are prominent in central nervous system (CNS). SIRT1, mainly nuclear, promotes mitophagy and mitochondrial biogenesis *via* deacetylation of LC3, FOXO3 and PGC-1α (Ng and Tang, 2013; Baeken, 2024). Its deficiency impairs autophagy and energy homeostasis, whereas its overexpression induces autophagy even under nutrient-rich conditions (Araki et al., 2004; Lee et al., 2008). SIRT3, the mitochondrial isoform of the sirtuins family, preserves mitochondrial integrity by directly regulating key enzymes of the TCA cycle and antioxidant defense systems, particularly during oxidative stress (Sidorova-Darmos et al., 2018). SIRT3 has been also shown to modulate mitophagy in specific disease contexts (Yu et al., 2017; Wang C. et al., 2019), and its overexpression alleviates neurological and mitochondrial dysfunction (Yang et al., 2024).

PARPs, particularly PARP1, are activated by DNA damage to catalyze ADP-ribosylation reactions that consume NAD^+^ and facilitate repair, thereby maintaining genomic integrity (Ray Chaudhuri and Nussenzweig, 2017). While moderate PARP activation supports cell survival, chronic or excessive activity depletes NAD^+^, causes mitochondrial depolarization, inhibits autophagy, and ultimately triggers cell death (Virág et al., 2013; Bai, 2015). PARP1 also modulates autophagy through AMPK–mTOR pathway and FOXO3a transcription factor (Muñoz-Gámez et al., 2009; Meng et al., 2018; Wang et al., 2018). In neurons, PARP-induced NAD^+^ depletion contributes to the pathogenesis of several neurodegenerative diseases (Mao and Zhang, 2022). Moreover, excessive PARP activation suppresses sirtuin activity either by depleting NAD^+^ or through transcriptional repression, thereby aggravating mitochondrial dysfunction (Cantó et al., 2013). Collectively, these findings underscore the therapeutic potential of targeting the PARP–SIRT interplay to preserve NAD^+^ homeostasis and protect against neurodegeneration.

CD38, an NADase whose activity increases with ageing, degrades NAD^+^ into ADP-ribose and cyclic ADP-ribose, making it a major contributor to the age-related decline in brain NAD^+^ levels (Camacho-Pereira et al., 2016; Tarragó et al., 2018). CD38-mediated NAD^+^ depletion reduces SIRT3 activity and impairs mitophagy, establishing a feed-forward loop that exacerbates mitochondrial dysfunction and energy failure (Camacho-Pereira et al., 2016). In neurons, another NADase, SARM1 (Sterile alpha and TIR motif-containing 1), acts as a metabolic sensor activated by axonal injury, cleaving NAD^+^ into nicotinamide and ADP-ribose to trigger axonal degeneration (Gerdts et al., 2015; Figley et al., 2021). This process contributes to pathological axonal pruning and neurodegeneration, whilst pharmacological inhibition of SARM1 confers neuroprotection in models of peripheral neuropathy and traumatic brain injury (Gerdts et al., 2015; Figley et al., 2021).

Collectively, these enzymes not only consume NAD^+^ but also modulate key metabolic and autophagy regulators such as AMPK and mTOR (Covarrubias et al., 2021; Wilson et al., 2023). Restoring NAD^+^ levels, frequently *via* precursors such as NMN or NR, enhance autophagic flux, sustain mitochondrial health, and improve neuronal survival in models of neurodegeneration (Yoshino et al., 2018; Lautrup et al., 2019; Xie et al., 2020). In summary, NAD^+^-consuming enzymes function as critical metabolic sensors that regulate neuronal autophagy, linking cellular energy metabolism to stress responses, protein turnover, and organelle maintenance—processes essential for neuronal function and survival.

## 3 Autophagy and NAD^+^ in neuronal homeostasis and neurodegenerative diseases

Defective autophagy is increasingly recognized as a major driver of neurodegeneration because impaired clearance of macromolecules (Menzies et al., 2015; Seranova et al., 2017; 2020; Palhegyi et al., 2019; Nixon and Rubinsztein, 2024) or damaged organelles (Narendra and Youle, 2024; Antico et al., 2025) disrupt neuronal homeostasis. Recent demonstration that the loss of autophagy depletes intracellular NAD^+^ levels suggests a role of autophagy in the maintenance of cellular NAD pool (Kataura et al., 2022; Sun et al., 2023). Decline in autophagic activity and NAD^+^ levels has been suggested as contributing factors to ageing and age-related neurodegenerative diseases that are marked by progressive neuronal cell death (Sedlackova and Korolchuk, 2020; Schmauck-Medina et al., 2022; Wilson et al., 2023; Kolotyeva et al., 2024) (Figure 1). In this section, we will discuss the interplay between autophagy and NAD^+^, and the therapeutic benefits of targeting the autophagy–NAD^+^ axis, in neurodegenerative diseases that are characterized by autophagy and NAD^+^ deficits (Table 1).

**TABLE 1:** Implications of autophagy-NAD^+^ axis in neurodegenerative diseases.

Autophagy impairment	NAD^+^ impairment	Bidirectional relationship	Therapeutic interventions
Alzheimer’s disease (AD)			
Impaired autophagy contributes to the build-up of Aβ plaques, hyperphosphorylated tau, and NFTs; mutations in presenilin-1 disrupt lysosomal acidification and Ca^2+^ homeostasis, impairing proteolysis; impaired mitophagy, mitochondrial damage, and oxidative stress are also linked to AD progression	Disrupted NAD^+^ metabolism contributes to mitochondrial dysfunction, increased oxidative stress, and impaired DNA repair in AD pathogenesis; NAD^+^-dependent enzymes, such as PARPs and SIRTs further implicated in AD progression	Boosting NAD^+^ levels by NMN, NR, or mitotherapy enhance autophagy and mitophagy *via* SIRT1 signaling, by promoting clearance of Aβ, damaged mitochondria, and hallmark proteins, ameliorating behavioural and cognitive impairments in AD models; overexpression of SIRT1/SIRT5, PARP mutation/inhibition restores mitochondrial function and autophagy, slowing AD progression	Pharmacological induction of autophagy by carbamazepine, trehalose, spermidine, and rapamycin, amongst others and boosting NAD^+^ levels by NAM, NR or NMN improve cognitive function in AD mouse models; SIRT1 activation or PARP1 inhibition is protective in cell and animal models of AD; ongoing clinical trials with NAD^+^ precursors, such as NR (NCT05617508; NCT04430517) or NMN (NCT05040321) are assessing dosing and treatment effects on brain metabolism and cognition
Parkinson’s disease (PD)			
Bioenergetic stress, ER stress, lipid dysregulation, and autophagy defects are linked to PD progression; α-synuclein abnormalities disrupts autophagy; PINK1/Parkin mutations impair mitophagy, increasing oxidative stress; mutations in LRRK2 causes autophagy-lysosomal dysfunction, promoting PD progression	Alterations in tryptophan metabolism, along with NAD^+^ levels, contribute to PD pathogenesis; NAD^+^-dependent enzymes such as PARPs and SIRTs further implicated in PD progression	PD-associated mutations in Parkin, PINK1 and FBXO7, impair mitophagy and lower NAD^+^ levels, likely *via* PARP activation; resveratrol restores autophagy, NAD^+^ levels, and energy homeostasis in PD *via* AMPK and SIRT1 activation	Pharmacological induction of autophagy by rapamycin, trehalose, felodipine, metformin, and 6-bio, amongst others and boosting NAD^+^ levels by NR, NMN, NAM, or β-NAD^+^ reduce neuronal loss and improve mitochondrial function and quality in PD cellular or animal models; PARP mutation/inhibition restores NAD^+^ levels mitochondrial function in cell and *Drosophila* models of PD; resveratrol enhance autophagy, NAD^+^ levels, and energy homeostasis in PD *via* AMPK and SIRT1 activation; phase I NR trials show safety, increased NAD^+^ levels, and mild clinical benefits with further trials ongoing (NCT05589766; NCT03568968; NCT05546567; NCT06162013)
Huntington’s disease (HD)			
Impaired autophagy drives accumulation of mHTT and neuronal toxicity; wHTT facilitates cargo recognition and autophagosome initiation; mHTT disrupts autophagosome transport, cargo loading, and lysosome fusion with toxic, non-ubiquitinated mHTT further resisting clearance; mitochondrial dysfunction also contributes to HD pathogenesis	Disrupted NAD^+^ metabolism and kynurenine contribute to HD pathogenesis; NAD^+^-dependent enzymes such as SIRTs, PARPs, and SARM1, further implicated in HD progression; oxidative stress and elevated ROS, exacerbate disease by driving excess NAD^+^ consumption through PARP and SIRT activation	Decline in NAD^+^ levels contribute to mitochondrial dysfunction, reduced antioxidant defense, and defective mitophagy in HD; NMNAT overexpression protects neurons by preventing SARM1-mediated NAD^+^ depletion and promotes autophagic clearance of mHTT aggregates, restoring neuronal function in HD models	Pharmacological induction of autophagy by rapamycin, rilmenidine, SMERs, and felodipine, amongst others improves progression in HD animal models; inhibition of PARP1 or SIRT2 show neuroprotective effects in HD mouse and *Drosophila* models; NAD^+^ boosting by NAM and niacin, are protective in HD animal models with further ongoing clinical trial with NR in HD patients (NCT06853743)
Niemann-Pick type C1 (NPC1) disease			
Impaired autophagy drives autophagosome build-up and abnormal lipid storage, contributing to NPC1 disease; mitochondrial dysfunction, including morphological defects, Ca^2+^ imbalance, ROS elevation, respiratory defects, depolarisation and reduced ATP levels further contributes to NPC1 disease pathology	Decline in NAD^+^ levels contribute to cell death in NPC1 disease	Decline in NAD^+^ levels impair autophagy, while boosting autophagy/mitophagy flux by small molecules attenuates both NAD^+^ depletion and cell death in NPC1 disease models	Pharmacological induction of autophagy by rapamycin, carbamazepine or celecoxib, or boosting NAD^+^ levels by NAM or NRH, is neuroprotective in NPC1 patient-iPSC-derived neuronal and other cellular models
Ataxia telangiectasia (AT)			
Impaired autophagy and mitophagy contribute to AT progress *via* perturbations in lysosomal acidification and mobilization	ATM-deficient cells, mice, and *C. elegans* display lower NAD^+^ levels accompanied by PARP1 hyperactivation and reduced SIRT1 activity	Restoring NAD^+^ levels in *Atm* ^*−/−*^ mice alleviates AT phenotypes, likely through enhanced mitophagy and DNA repair	Boosting NAD^+^ levels by NR or NMN prevents neurodegeneration, improves motor function, and extends lifespan in animal models; NR trials shows better coordination, eye movements and ataxia scores
Autism spectrum disease (ASD)			
Impaired autophagy and mitophagy contribute to ASD progress; *Atg7* deletion in the microglia or forebrain interneurons causing ASD-like social deficits and repetitive behaviours	Decline in NAD^+^, NADH, and ATP levels contributes to ASD pathogenesis; impaired tryptophan metabolism further contributes to ASD progress	Not known	Not known

References are available in text.

### 3.1 Alzheimer’s disease

Alzheimer’s disease (AD) is the most prevalent neurodegenerative disorder. It is associated with cognitive impairment and behavioral symptoms due to progressive neuronal loss (Knopman et al., 2021; Zhang et al., 2024). Familial AD is driven by autosomal dominant mutations in amyloid precursor protein (*APP*) or presenilin (*PSEN1* or *PSEN2*) genes, which lead to increased β-amyloid (Aβ) production. In contrast, sporadic AD that accounts for over 95% of cases, arises from a multifactorial interplay of genetic risk factors, ageing, metabolic stress, and mitochondrial dysfunction (Dorszewska et al., 2016). AD is pathologically defined by the accumulation of Aβ plaques and tau neurofibrillary tangles (NFTs) in the brain (Knopman et al., 2021; Zhang et al., 2024). Increasing evidence suggests crosstalk between Aβ and tau that contributes to cellular dysfunctions and neurotoxicity (Zhang et al., 2021). A complex interplay of disruption in synaptic homeostasis and cellular clearance pathways like autophagy has been suggested in AD pathogenesis (Knopman et al., 2021; Zhang et al., 2021; 2024).

Autophagy dysfunction is considered a major contributing factor in neuronal vulnerability to genetic and environmental factors underlying AD. Autophagy has been suggested to play a role in the generation of Aβ, *via* processing of the amyloid precursor protein (APP) by presenilin-1 inside the autophagosomes. Aβ either undergoes autophagic degradation or is secreted into extracellular space by autophagy (Yu W. H. et al., 2005; Nilsson et al., 2013; Tian et al., 2013). Moreover, tau is also degraded by autophagy (Wang et al., 2009; Krüger et al., 2012; Lee et al., 2013; Neu et al., 2025). Defective autophagic clearance leads to the accumulation of Aβ and hyperphosphorylated tau, contributing to synaptic dysfunction and neuronal cell death (Nixon, 2024; Nixon and Rubinsztein, 2024). Failure in autophagy has been evidenced by the accumulation of autophagic vacuoles in AD patient brains, dystrophic neurites in mouse models and iPSC-derived cortical neurons, indicative of a block in autophagic flux that is suggested to arise from impaired lysosomal proteolysis (Boland et al., 2008; Nixon and Yang, 2011; Piras et al., 2016; Hung and Livesey, 2018). Mutations in presenilin-1 mediate autophagic dysfunction by impairing lysosomal acidification and Ca^2+^ homeostasis, thereby disrupting lysosomal proteolytic activity (Lee et al., 2010; 2015; Lee J.-H.et al., 2020). Mutant tau can also cause buildup of autophagosomes by preventing axonal transport *via* destabilization of microtubules (Rodríguez-Martín et al., 2013; Butzlaff et al., 2015; Cario and Berger, 2023). Furthermore, impairment of mitophagy has been demonstrated in AD patient brain and iPSC-derived neurons, and in cell and animal models (Cummins et al., 2019; Fang et al., 2019b; Mary et al., 2023). This could lead to the accumulation of damaged mitochondria and oxidative stress that are involved in the progression of AD (Wang et al., 2020; Ionescu-Tucker and Cotman, 2021). In addition, multiple lines of evidence suggest disruption in NAD^+^ metabolism as a contributing factor in AD (Lautrup et al., 2019; Wang et al., 2021). Decreased NAD^+^ and NADH levels, observed in both ageing and AD mouse brains and neuronal cultures, are linked to mitochondrial dysfunction and oxidative stress (Abeti et al., 2011; Liu D. et al., 2013; Hou et al., 2018; 2021; Lautrup et al., 2019; van der Velpen et al., 2021). NAD^+^-consuming enzymes, such as PARPs and SIRTs, are also implicated in AD—although SIRT activity is reduced in AD, PARP is activated that could drive NAD^+^ depletion (Strosznajder et al., 2012; Mehramiz et al., 2023). Together, these findings highlight a pathological link between autophagy and mitochondrial dysfunction, along with NAD^+^ deficits, in AD pathogenesis (Bhatia and Sharma, 2021).

Therapeutic strategies involving upregulation of autophagy and NAD^+^ levels have been shown to improve neuronal health and cognitive outcomes in AD animal models (Menzies et al., 2017; Dölle and Tzoulis, 2025). Pharmacological autophagy inducers, such as carbamazepine, trehalose, spermidine, and rapamycin or its analog temsirolimus, amongst others, have been shown to enhance the clearance of Aβ plaques and tau tangles, and ameliorate disease phenotypes including cognitive deficits, in various AD mouse models (Caccamo et al., 2010; Rodríguez-Navarro et al., 2010; Spilman et al., 2010; Majumder et al., 2011; Schaeffer et al., 2012; Du et al., 2013; Li et al., 2013; Ozcelik et al., 2013; Jiang et al., 2014a; 2014b; Portbury et al., 2017; Freitag et al., 2022). Supplementation with NAD^+^ precursors, such as NAM, NR or NMN, restores NAD^+^ levels and also prevents neurodegenerative pathology and cognitive decline in AD mouse models (Green et al., 2008; Gong et al., 2013; Liu D. et al., 2013; Yao et al., 2017; Hou et al., 2018; 2021; Rehman et al., 2021; Ma et al., 2024; Xiong et al., 2024). Interestingly, some studies have revealed potential crosstalk between these pathways. For instance, NMN promotes the clearance of hyperphosphorylated tau by enhancing autophagy (Ma et al., 2024), also lowers Aβ plaques (Yao et al., 2017), to ameliorate behavioural and cognitive impairments in AD mice. Likewise, NMN and NR prevent cognitive decline and proteotoxicity in *C. elegans* models of AD by inducing mitophagy (Sorrentino et al., 2017; Fang et al., 2019b). Beyond NAD^+^ replenishment, mitotherapy—introducing healthy mitochondria into defective neuronal cells—has been shown to activate autophagy *via* the NAD^+^-dependent SIRT1 signalling pathway to enhance the clearance of Aβ and damaged mitochondria, and improve cognitive function, in a mouse model of AD (Yang et al., 2023). Additionally, overexpression of SIRT1 or SIRT5, genetic mutation of *Parp*, or inhibition of PARP1 is protective in cell and animal models of AD (Qin et al., 2006; Min et al., 2010; Abeti et al., 2011; Martire et al., 2016; Wu et al., 2021; Yu et al., 2021).

There have been multiple clinical trials with NAD^+^ precursors in AD (Dölle and Tzoulis, 2025). Oral administration of NR in healthy individuals elevates neuronal NAD^+^ levels and lowers biomarkers related to neurodegenerative pathology (Vreones et al., 2023). However, clinical trials in AD with NR or NAM have not yielded any major cognitive improvements (Orr et al., 2024; Grill et al., 2025). Ongoing trials are evaluating the optimal dose and treatment period on brain metabolism and cognitive function, amongst other parameters, with NR (NCT05617508; NCT04430517) or NMN (NCT05040321). Overall, the interplay between autophagy and NAD^+^ metabolism, and their convergence on key cellular processes such as mitochondrial homeostasis and neuronal survival, highlight the therapeutic promise of targeting the autophagy–NAD^+^ axis in AD.

### 3.2 Parkinson’s disease

Parkinson’s disease (PD) is the second most prevalent neurodegenerative disease worldwide. It is clinically characterized by motor symptoms including resting tremor, bradykinesia, rigidity, and postural instability, as well as a broad spectrum of non-motor features including cognitive changes (Poewe et al., 2017; Bloem et al., 2021). Pathologically, PD is marked by the progressive degeneration of dopaminergic neurons in the substantia nigra pars compacta and the accumulation of Lewy bodies that are mainly composed of aggregated α-synuclein (Kalia and Kalia, 2015; Poewe et al., 2017). Lewy body pathology in PD not only contributes to dopaminergic neuronal loss but also disrupts dopamine neurotransmission by impairing intracellular transport (Kalia and Kalia, 2015; Aarsland et al., 2021; Calabresi et al., 2023). The selective vulnerability of dopaminergic neurons in PD has been linked to mitochondrial dysfunction (Borsche et al., 2021; Henrich et al., 2023). Multiple cellular stress pathways aid to mitochondrial abnormalities, including bioenergetic disruption, ER stress, altered lipid metabolism, and autophagy malfunction (Lynch-Day et al., 2012; Van Laar and Berman, 2013; Gómez-Suaga et al., 2018; Zambon et al., 2019; Flores-Leon and Outeiro, 2023).

Impairment in autophagy, including chaperone-mediated autophagy (CMA), in PD contributes to α-synuclein aggregation, mitochondrial dysfunction, and neuronal loss (Cuervo et al., 2004; Lynch-Day et al., 2012; Menzies et al., 2015; Hou et al., 2020). PD is a multigenic disorder, and mutations in multiple PD-associated genes deregulate autophagy at distinct stages, including the selective autophagy process of mitophagy as well as CMA (Lynch-Day et al., 2012; Gan-Or et al., 2015). α-synuclein gene multiplication inhibits autophagosome biogenesis (Winslow et al., 2010; Decressac et al., 2013), Lewy bodies containing α-synuclein prevent autophagosome maturation (Tanik et al., 2013), whilst α-synuclein point mutations (A53T or A30P) impair CMA (Cuervo et al., 2004; Martinez-Vicente et al., 2008). Mutations in mitophagy-related genes, encoding for PINK1 and Parkin, further compromise mitochondrial clearance. Loss-of-function mutations in PINK1 and Parkin impair the recognition and degradation of damaged mitochondria, thereby increasing oxidative stress and energy failure (Pickrell and Youle, 2015; Narendra and Youle, 2024). Mutations in LRRK2 is suggested to cause autophagy and lysosomal dysfunction, as well as disruption of CMA (Orenstein et al., 2013; Madureira et al., 2020).

Alterations in metabolic pathways also contribute to the pathogenesis of PD. One such example is the kynurenine pathway of tryptophan metabolism, which influences NAD^+^ levels and is implicated in PD (Lim et al., 2017; Castro-Portuguez and Sutphin, 2020). Multiple studies in genetic and neurotoxin-induced models have demonstrated impairment in NAD^+^ metabolism and decrease in NAD^+^ levels in PD, along with the role of NAD^+^ depletion in dopaminergic neurodegeneration (Schöndorf et al., 2018; Lundt and Ding, 2021; Pérez et al., 2021). For instance, PD-associated mutations in Parkin, PINK1 and FBXO7, all of which impair mitophagy, reduce cellular NAD^+^ levels that is suggested *via* PARP activation (Burchell et al., 2013; Lehmann et al., 2016; 2017; Delgado-Camprubi et al., 2017). Moreover, NAD^+^-consuming enzymes such as PARPs and SIRTs have also been implicated in PD, further underscoring the involvement of NAD^+^-dependent mechanisms (He et al., 2022; Lengyel-Zhand et al., 2022).

Therapeutic strategies involving autophagy induction and NAD^+^ restoration in PD models have demonstrated promising neuroprotective effects, including amelioration of motor deficits and neuropathological phenotypes (Menzies et al., 2017; Pérez et al., 2021; Sanchez-Mirasierra et al., 2022; Dölle and Tzoulis, 2025). Pharmacological agents inducing autophagy, such as rapamycin, trehalose, felodipine, metformin, and 6-Bio, amongst others, have been shown to alleviate disease phenotypes in mouse models of PD (Malagelada et al., 2010; Liu K. et al., 2013; Bai et al., 2015; Suresh et al., 2017; Ryu et al., 2018; Pupyshev et al., 2019; Siddiqi et al., 2019). Likewise, supplementation with NAD^+^ precursors, such as NR, NMN, NAM, and β-NAD^+^, have been shown to improve mitochondrial bioenergetic function and exert cytoprotective effects in cellular and animal models of PD (Jia et al., 2008; Lu et al., 2014; Lehmann et al., 2016; 2017; Zou et al., 2016; Schöndorf et al., 2018; Shan et al., 2019; Pérez et al., 2021). For instance, NR administration rescues mitochondrial dysfunction in PD patient iPSC-derived neurons and prevents the decline of motor ability and age-dependent loss of dopaminergic neurons in GBA-PD *Drosophila* model by increasing NAD^+^ levels (Schöndorf et al., 2018). Moreover, modulating the activity of NAD^+^-consuming enzyme, such as by PARP inhibition or mutation, restores NAD^+^ levels and rescues mitochondrial dysfunction in cell and *Drosophila* models of PD (Lehmann et al., 2016; 2017; Delgado-Camprubi et al., 2017). Amongst others, resveratrol has been shown to improve autophagy, NAD^+^ levels and energy homeostasis in PD patient fibroblasts *via* activation of AMPK and SIRT1 (Ferretta et al., 2014). Randomised phase I clinical trials with NR in 20–30 PD patients have reported no safety concerns, elevation in brain and blood NAD^+^ levels, along with mild clinical improvement (Brakedal et al., 2022; Berven et al., 2023). Clinical trials involving larger patient cohort with NR (NCT05589766; NCT03568968; NCT05546567; NCT06162013) are currently ongoing. Overall, these studies highlight the therapeutic potential of targeting the autophagy–NAD^+^ axis in PD.

### 3.3 Huntington’s disease

Huntington’s disease (HD) is a progressive, autosomal dominant neurodegenerative disorder caused by a CAG trinucleotide expansion in the Huntingtin (*HTT*) gene (Bates et al., 2015). This expansion results in an abnormally extended polyglutamine (PolyQ) tract in the huntingtin protein (HTT), producing a mutant form (mHTT) with toxic gain-of-function properties (Zoghbi and Orr, 2000). The accumulation of mHTT aggregates within neurons is a pathological hallmark of HD and contributes to widespread neuronal dysfunction and degeneration (Yamamoto et al., 2000). The number of CAG repeats is strongly associated with disease characteristic—larger expansions correlate with earlier onset and increased severity. Alleles with more than 40 CAG repeats are fully penetrant and lead to HD development, and typically with more than 60 CAG repeats in early-onset. Intermediate alleles (36–39 CAG repeats) show reduced penetrance, while alleles with fewer than 36 CAG repeats are considered non-pathological (Zoghbi and Orr, 2000; Bates et al., 2015). Clinically, HD presents with a triad of progressive motor dysfunction, cognitive decline, and psychiatric disturbances, progressively worsening into dementia (Peavy et al., 2010; Roos, 2010). The motor symptoms are primarily attributed to selective degeneration of medium spiny neurons in the striatum, which precedes cortical neuronal loss (Zoghbi and Orr, 2000; Bates et al., 2015; Oh et al., 2022).

A critical cellular process disrupted in HD is autophagy, which is the predominant intracellular clearance route for mHTT (Sarkar and Rubinsztein, 2008; Menzies et al., 2015). Wild-type HTT functions as a scaffold protein, facilitating cargo recognition and autophagosome initiation (Ochaba et al., 2014; Rui et al., 2015). In contrast, mHTT impairs autophagy at multiple stages—disrupting autophagosome transport, hindering cargo loading, and blocking fusion with lysosomes (Martinez-Vicente et al., 2010; Wong and Holzbaur, 2014; Oh et al., 2022; Pircs et al., 2022). Studies in various HD models have elucidated how dysregulated autophagy contributes to mHTT accumulation and neuronal toxicity (Martin et al., 2015; Menzies et al., 2015). Moreover, mHTT can undergo conformation-dependent recognition by selective autophagy that correlates with their degradation and toxicity. A toxic mHTT protein lacking Lys63 polyubiquitination and p62 interaction, which are essential for aggrephagy (degradation of protein aggregates by autophagy), slow autophagic clearance due to its resistance to this selective autophagy process (Fu et al., 2017).

Apart from defective autophagy, mitochondrial dysfunction and disrupted NAD^+^ metabolism are pathological features in HD that reflect impairment in cellular energy homeostasis (Lundt and Ding, 2021; Sharma et al., 2021). This decline in NAD^+^ levels in HD is linked to mitochondrial dysfunction, weakened antioxidant defence, and aberrant mitophagy (Xie et al., 2020), which could be arising due to autophagy defect. Hyperactivation of kynurenine pathway as well as NAD^+^-consuming enzymes, such as SIRTs, PARPs, and SARM1, are also implicated in the pathogenesis of HD (Lloret and Beal, 2019; Xie et al., 2020; Lundt and Ding, 2021). Oxidative stress and elevated ROS, commonly reported in HD (Kim et al., 2021; Sharma et al., 2021), can further activate PARPs and SIRTs that accelerate NAD^+^ consumption. As a potential therapeutic intervention, pharmacological inhibition of PARP1 or SIRT2 demonstrate neuroprotective effects in HD mouse and *Drosophila* models (Pallos et al., 2008; Cardinale et al., 2015; Paldino et al., 2017). In a study involving injury-induced axonal degeneration where NAD^+^ levels were decreased, overexpression of the NAD^+^ biosynthetic enzyme NMNAT1 is shown to protect mouse dorsal root ganglion neurons against axonal damage by preventing SARM1-mediated NAD^+^ depletion (Sasaki et al., 2016). Nmnat overexpression also restores neuronal function in a *Drosophila* model of HD where the neuroprotective effects are attributed to autophagic removal of mHTT aggregates (Zhu et al., 2019).

Given the interdependence between autophagy, mitochondrial integrity, and NAD^+^ metabolism, numerous studies have explored therapeutic strategies targeting these pathways. Pharmacological inducers of autophagy, such as rapamycin, rilmenidine, SMERs, and felodipine, amongst others, have been shown to enhance autophagic flux, facilitate mHTT clearance, and alleviate disease phenotypes in various HD animal models (Ravikumar et al., 2004; Sarkar et al., 2007; Williams et al., 2008; Rose et al., 2010; Menzies et al., 2017; Siddiqi et al., 2019). Likewise, supplementation with NAD^+^ precursors, such as NAM and niacin, are protective in HD animal models (Pallos et al., 2008; Hathorn et al., 2011; Xie et al., 2020; Dölle and Tzoulis, 2025). Recently, a clinical trial has been launched to study the efficacy and safety of NR in HD patients (NCT06853743). Overall, targeting the autophagy–NAD^+^ axis has demonstrated therapeutic benefits in HD models.

### 3.4 Niemann-Pick type C1 disease

Niemann-Pick Type C1 (NPC1) disease is a rare autosomal recessive lysosomal storage disorder primarily caused by mutations in the *NPC1* gene, which encodes a polytopic transmembrane cholesterol transporter located on the lysosomal membrane (Li et al., 2017). Loss of NPC1 function impairs intracellular lipid trafficking, leading to the accumulation of lipids, particularly cholesterol and sphingolipids, in the late endosomes and lysosomes (Lloyd-Evans et al., 2008; Patterson et al., 2017). Clinical presentations of NPC1 disease are highly heterogenous and depend on the age of onset, although the disease is typically characterised by progressive neurodegenerative dementia and hepatosplenomegaly (Vanier et al., 1996; Las Heras et al., 2023). The *NPC1* gene is highly conserved across eukaryotes, and thus various disease models have provided key insights into the pathophysiology of NPC1 disease (Fog and Kirkegaard, 2019; Lee S.-E. et al., 2020), showing that defective lipid homeostasis causes cellular degeneration through mechanisms involving impaired autophagy, mitochondrial dysfunction, and more recently, NAD^+^ decline (Sarkar et al., 2013; Maetzel et al., 2014; Vilaça et al., 2014; Kataura et al., 2022; 2024).

Impaired autophagic flux in NPC1 disease is characterised by the accumulation of autophagosomes due to defective SNARE machinery, which is required for the fusion of autophagosomes with late-endosomes or lysosomes (Sarkar et al., 2013). Additionally, reduced VEGF levels leading to abnormal sphingosine accumulation have been implicated in autophagy dysfunction and neuronal loss in NPC1 disease (Lee et al., 2014). A recent study identified that lysosomal cholesterol accumulation leads to hyperactivation of mTORC1 (Davis et al., 2021), which can repress autophagy by inhibiting the autophagy initiator ULK1 complex. These findings indicate that multiple molecular mechanisms underlie autophagy dysfunction in NPC1 disease. Nonetheless, stimulating autophagy with rapamycin, carbamazepine or celecoxib restore autophagic flux and improve cell viability in NPC1 patient iPSC-derived neurons (Maetzel et al., 2014; Kuo et al., 2015; Kataura et al., 2024).

Mitochondrial dysfunction is also evident in NPC1 disease. In NPC1-deficient cells, cholesterol is significantly elevated in mitochondrial membranes, which is associated with various mitochondrial abnormalities such as morphological defects, elevated Ca^2+^ levels, increased ROS production, reduced respiratory capacity, mitochondrial depolarisation and decreased ATP levels (Yu W. et al., 2005; Woś et al., 2016; Casas et al., 2023; Kataura et al., 2024). Mitochondrial dysfunction can trigger apoptosis and influence other regulated cell death pathways including necroptosis, both of which have been implicated in NPC1 pathogenesis (Erickson and Bernard, 2002; Cougnoux et al., 2016; Zareba et al., 2024). Given that autophagy plays a critical role in mitochondrial quality control *via* mitophagy, impaired autophagy and aberrant mitochondrial cholesterol may act synergistically to disrupt mitochondrial homeostasis and promote cell death in NPC1 disease.

Recently, declining NAD^+^ levels have been identified as a key mediator of cell death in NPC1 disease and are closely associated with autophagy dysfunction (Kataura et al., 2022; 2024). In NPC1-deficient cells, both NAD^+^ and NADH levels are significantly reduced whilst boosting NAD^+^ levels by supplementing NAD^+^ precursors, such as NAM or NRH, restores cell survival. Notably, enhancing autophagy/mitophagy flux using small molecules attenuates both NAD^+^ depletion and cell death, suggesting that NAD^+^ loss occurs downstream of impaired autophagy and serves as a trigger for cell death––consistent with the previous findings in *ATG5* ^−/−^ autophagy-deficient cells (Kataura et al., 2022; 2024; Sun et al., 2023). These results support the concept that the autophagy–NAD^+^ axis could be a new therapeutic target in NPC1 disease.

Currently, two drugs—Miglustat and Arimoclomol—have been approved for NPC1 treatment. Miglustat, the first approved drug, is a glucosylceramide synthase inhibitor that reduces glycosphingolipids accumulation and remained the only available treatment for 15 years (Pineda et al., 2018) Similarly, the cholesterol-lowering agent, 2-Hydroxypropyl-β-cyclodextrin (HPβCD) has shown promise in clinical trials for NPC1, although further validation is needed (Hastings et al., 2022). In 2024, the FDA approved Arimoclomol as part of a combination therapy with Miglustat. Arimoclomol is thought to enhance lysosomal function by upregulating heat shock proteins and promote cell survival under lysosomal stress (Mengel et al., 2021). As this drug was effective in patients who received Miglustat as their background treatment (Mengel et al., 2021), combination therapies that act *via* multiple mechanisms appear promising for NPC1 disease. Notably, stimulating autophagy, which is sufficient to rescue cell death in NPC1-deficient cells, was unable to alleviate cholesterol accumulation. Likewise, HPβCD did not improve autophagic function (Sarkar et al., 2013). Taken together, it is possible that a dual approach—combining lipid-lowering agents with drugs targeting the autophagy–NAD^+^ axis—may offer an optimal disease-modifying strategy for NPC1 disease.

### 3.5 Other neurological diseases

Ataxia-telangiectasia (A-T) is a rare, autosomal recessive genetic disorder, characterized by progressive cerebellar ataxia, telangiectasia and immunodeficiency (Collyer and Rajan, 2024). A-T is caused by mutations in ataxia telangiectasia-mutated (*ATM*) gene, which encodes for ATM kinase that regulates of double-strand DNA break (DSB) signalling and stress responses (Lee and Paull, 2021). ATM deficiency is associated with impairment in DNA damage response, mitochondrial dysfunction and oxidative stress (Valentin-Vega et al., 2012; Fang et al., 2016; Stagni et al., 2018; Lee and Paull, 2021; Leeson et al., 2024). ATM is suggested to undergo autophagic degradation and aid in autolysosome formation, whereas its loss deregulates autophagy and mitophagy possibly *via* perturbations in lysosomal acidification and mobilization (Fang et al., 2016; Stagni et al., 2020; Sunderland et al., 2020; Cheng et al., 2021; Hwang et al., 2023). Additionally, ATM-deficient cells, mice and *C. elegans* display lower NAD^+^ levels (Fang et al., 2016; Yang et al., 2021). This was found to be associated with increased PARP1 and reduced SIRT1 activity in ATM-deficient cells (Fang et al., 2016). Boosting NAD^+^ levels *via* supplementation with NAD^+^ precursors, such as NR or NMN, prevents neurodegeneration, improves motor function, and extends lifespan in animal models (Fang et al., 2016; Yang et al., 2021). Alleviation of A-T phenotypes by restoring NAD^+^ levels in *Atm* ^*−/−*^ mice are suggested to be *via* upregulation of mitophagy and DNA repair (Fang et al., 2016; Yang et al., 2021). Clinical trials with NR in a small cohort of A-T patients have shown improvements in motor coordination, eye movements and ataxia scores (Veenhuis et al., 2021; Steinbrücker et al., 2023; Presterud et al., 2024). Overall, these findings suggest NAD^+^ supplementation as a promising therapeutic strategy for A-T.

Autism spectrum disorder (ASD) is a multifaceted neurodevelopmental condition characterised by difficulties in social communication and repetitive behaviours (Lord et al., 2020). Autophagy and mitochondrial dysfunction, along with oxidative stress, have been reported in ASD (Essa et al., 2013b; Deng et al., 2021). Impairment in autophagy, suggested to be arising from mTOR activation, is seen in autistic children as well as in rat, mouse and cellular experimental models of ASD (Tang et al., 2014; Huber et al., 2015; Zhang J. et al., 2016; Lieberman et al., 2020; Deri et al., 2025; Ham et al., 2025). Perturbations in the expression and phosphorylation of several autophagy-related proteins has been found *via* multi-omics approach in ASD mouse brain (Deri et al., 2025). Moreover, deletion of *Atg7*, an essential autophagy gene, in the microglia or forebrain interneurons in mice leads to ASD-like social behavioural impairments and repetitive behaviours (Kim et al., 2017; Hui et al., 2019). In addition, decreased levels of NAD^+^, NADH and ATP are found in patients with autism (Adams et al., 2011; Essa et al., 2013a; Launay et al., 2023). Tryptophan metabolism, which generates NAD^+^ and several neuroactive intermediates, is reported to be impaired in ASD, as evidenced by low tryptophan levels in autistic children (Kałuzna-Czaplinska et al., 2010; Essa et al., 2013b), whilst other studies found no change (Almulla et al., 2023; Launay et al., 2023). More studies are warranted to determine the therapeutic benefits of autophagy–NAD^+^ axis in experimental models of ASD.

## 4 Conclusions and prospects

Autophagy and NAD^+^ play a pivotal role in the regulation of numerous cellular processes, including energy metabolism, genomic maintenance, stress resistance, cell survival. Although emerging evidence have underscored the importance of the reciprocal relationship between autophagy and NAD^+^ metabolism, their crosstalk in the progression of neurodegenerative diseases by maintaining cellular, mitochondrial and bioenergetic homeostasis is not completely understood. To bridge this knowledge gap, future research should aim to elucidate the underlying mechanisms of this bidirectional interaction and its impact on neuronal health and the neurodegenerative process. Importantly, most current findings have been derived from conventional *in vitro* and animal models, which often fail to fully recapitulate human neuronal complexity and disease pathology. Therefore, there is an urgent need to employ more physiologically relevant systems—such as iPSC-derived cerebral organoids—to better model the intricate dynamics of the autophagy–NAD^+^ relationship in a disease-specific context.

From a therapeutic standpoint, pharmacological induction of autophagy and enhancement of NAD^+^ levels using precursors such as NAM, NMN, NR, and NRH have individually demonstrated neuroprotective effects in preclinical studies. These interventions have been associated with improved mitochondrial function, reduced protein aggregation, and enhanced neuronal survival. Future therapeutic interventions could focus on modulating NAD^+^ metabolism and autophagy to restore cellular function and mitigate the effects of neurodegeneration.
